# “Barsati,” or Atrophic Carcinoma

**Published:** 1886-10

**Authors:** Richard W. Burke

**Affiliations:** Cawnpore, India


### ART. XIX.—"BARSATI," OR ATROPHIC CARCINOMA.

BY RICHARD W. BURKE, V. S., A. V. D.
*Cawnpore, India.*

From 1838 to 1851 numerous essays, papers, and other contributions were published on this subject of *Barsati*, especially those which came from the pens of Army veterinarians serving at that time in India; and numerous were the theories then propounded as to its nature. It was not until 1873, however, that Spooner Hart, of Calcutta, an enthusiastic veterinarian and accurate observer, published his essay in a series of excellent papers contributed to *The Veterinarian*, far in advance of all preceding descriptions, and possessing that touch of critical appreciation which alone distinguishes a practical surgeon and clinical observer. But in recent years the opinion has been gradually coming into prominence that the clinical characteristics of disease have not the value as an element of inquiry which many would assign to them; that they are not, singly, the be-all and end-all of our pathological problems. Clinical knowledge, whether viewed as an art or a profession, should ever be agreeably associated with the other departments that are interesting in science and pleasurable in inquiry.

So much has already been written by way of contribution to our knowledge of this disease that, important as the subject is, it may be considered to have received already a fair share of attention; the pathology of it, however, cannot, perhaps, be dwelt upon too much, if we would stimulate a practical inquiry into its causation and treatment.

It is not necessary to remark that my observations led me to state as early as 1880* that this disease was cancer of the horse; and I believe no other writer has preceded me in this conclusion, so far as my knowledge of its literature has shown.

These observations refer to a few additional branches of inquiry set forth here, and I believe they may not be devoid of interest as supplying a continuation of the history of the disease; while, at the same time, it will be of some importance on account of its nomenclature and pathology, Barsati being confounded in India with many other diseases, some of which are sufficiently characteristic, while others may be said to possess only a casual resemblance to it. It is therefore necessary to have as clear a view of the disease under consideration as possible, which can only be obtained by comparing its characteristics as they occurred to various observers in separate localities, in different years and seasons, and under varied conditions of climate and treatment. Barsati flourishes in the low-lying parts of India where heat and moisture are combined. It is seldom or never heard of on the hills or in dry, elevated localities. Mr. Oliphant has recently observed that taking horses, the subject of Barsati, to the hills, brings about a speedy improvement. And the Collective Investigation Committee in England, have been lately engaged in an inquiry into the relative prevalence of human cancer, which so far establishes a similar explanation in regard to geographical and hygrometric distribution. Dr. Henry Butlin † writes, that "High and dry localities are unfavorable to the occurrence of cancer, and that the disease flourishes chiefly in low, flat parts of the country" (England), "which are covered by alluvium, and watered by many streams that are subject to frequent floods."

Dr. Walshe ‡ writes: "Certain regions of the globe are peculiarly exempt from the ravages of cancer. But is this exemption to be really referred to the special influence of climate

*Veterinary Journal, 1880.

†Brit. Med. Journal, July 11, 1885, p. 53.

‡Treatise on Cancer, by R. Mitchell, 1879.

or to some concomitant condition?" Dr. Lyford,* of America, writing on Barsati, remarks: "It seems to be confined to the neighborhood of Minneapolis and St. Paul, with the exception of a few cases, which migrate each year. Under these circumstances the question naturally arises, Why should this disease appear in two localities so far distant from each other as India and Minnesota, and under such different circumstances, and without the least sign of the disease in any of the intermediate localities?"

Jessett † says "Cancer is more ripe in *low-lying damp situations*" where cancer is more prevalent in England, such as we have found in this country, and Dr. Lyford has noted in America. Much stress has, from the first, been laid on the effects of wet weather, because it was observed that almost all the cases of Barsati were aggravated during the rains. In spite of the stress laid on this point, it was shown by the records of the late Bengal Studs that by far the largest number of cases of Barsati was actually admitted for treatment long before the commencement of the rainy season, ‡ and the months of March, April, and May are mentioned as showing the largest percentage of admissions.

A wet climate may predispose to the disease by enfeebling the animal's constitution, so that the tissues are rendered deficient in tone and nutritive functions by the sudden excess of moisture in the atmosphere, and by its direct injurious action on the local tissues, as seen in many forms of skin disease. Cutaneous disorders of a somewhat obstinate and chronic character are always found prevalent during the wet weather, from hyperaction of the skin, and which no amount of local treatment will generally remove. It has been observed that heat and moisture are favorable to the growth of vegetable fungi, which are the cause of Barsati sores becoming aggravated during the rains. If this be a sufficient explanation, how does it happen that simple sores are similarly aggravated during wet weather in India? The notion of parasitic action is contradicted posi-

---

*Veterinary Journal, May, 1886, pp. 379-81.

†Brit. Med. Journal, April 26, 1884.

‡The rains do not begin till the latter part of June, and end in September. The term Barsati is therefore, a misnomer.

tively by the fact of wet weather proving hurtful alike to Barsati as well as to all simple sores.

Here, then, we perceive the opening of a most interesting and fruitful line of pathological inquiry, not yet ever seriously attempted, namely, the deleterious effects of heat and moisture upon newly injured tissues.

I have in the past brought forward facts, both clinical and pathological, to show the relationship that exists between human cancer and Barsati in all the main features. These facts, while they might serve to prove that fungi exist in sores exposed to the air, are wholly opposed to the idea that the peculiar cell changes occurring in this disease are necessarily caused by such fungi. Indeed, to imagine that fungi met with in sores once exposed to the action of the air would explain the morbid changes, must be regarded as rather sanguine. There is nothing in the history of the disease, nor in the results of its treatment, to show that its cause is likely to be parasitic. Experiments by Professor Uskoff, of Cronstadt, on dogs, show that marked necrotic changes in tissues in many cases are unattended by the appearance of organisms in even the smallest number, and have therefore led to the conclusion that the universal dependence of structural changes on parasitic organisms, maintained by some persons, is incorrect. The appearance of organisms in some cases he ascribes to the possibly imperfect removal of them from the liquids used, or to the circumstance that, by their mechanical injury to the tissues, the power of resistance was lowered, which allowed the growth of the organisms. It will thus be seen that his investigations entirely support the position maintained by several of the foremost pathologists of the day. Drs. Lewis and Cunningham, of Calcutta, have already shown how, similarly, the growth of fungi in Delhi-boil may occur as the result of the tissues being steeped in chromic acid and chromate of potash solutions. Quite recently Dr. Fenwick has found that in tissues hardened in a four per cent. solution of cocain, a peculiar mould fungus always develops, whose presence is accidental and due entirely to the process of hardening the tissue.*

*Lancet, 1885.

Parasitic organisms frequently develop also in specimens stained in carmine solution.*

Warden and Waddell † have recently experimented with the Arbus poison. Hypodermic injections were made, mainly on cats and fowls, in order to determine whether a general parasitic condition was necessarily associated with the toxic action of the seeds. From the general results of their experiments the authors conclude that the presence of organisms at the seat of injection is purely accidental, and that these develop from the air after the injection. Professor Ponfick has demonstrated that organisms of various kinds are always present in tissues after the injection of turpentine and some other chemical irritants.‡

We cannot directly prove that parasites here precede the disease changes; indeed, all experience teaches the reverse; yet from theoretical reasoning we must conclude that such must be the case. But it seems to me that the proofs of clinical experience are of even greater consequence than any theoretical conclusions. If the parasites were the cause the disease would be communicable by inoculation. Yet, experiment has long decided in favor of the non-communicable nature of the disease. Those who favor the view that Barsati is of a parasitic origin, give us no proof in support of their view, which, to some extent, converts the morbid process into a sort of skin "sepsis" and contagious would thus differ from simple disease only in degree; since all local virulent action is absent in this disease, how can we distinguish between a simple and "infective" form of disease? May the one at any time become the other, and, if so, in obedience to what law? Those who support the "fungus" origin of Barsati leave this matter very much an open question, as indeed was unavoidable, for we do not know enough about the other varieties of this disease. It is remarkable that a series of inoculation experiments—scarifications and hypodermic and intravenous injections—conducted by myself as well as by other Veteri-

---

*Brit. Med. Journ., July 18, 1885.

† 'The Non-parasitic Nature of Arbus Poison,' by C. H. Warden and L. A. Waddell, M. B., Calcutta, 1884.

‡ Virchow's Archiv., 1881.

nary-Surgeons should have been followed by no results, either local or constitutional. Indeed, judged by the verdict of common experience, the disease cannot be communicated, and is therefore not contagious.

If the analogy of parasitic action holds good between cancer of man and Barsati in the horse, we must assume that in every instance the disease has been "caught." But experience proves this to be correct in respect of neither. Thus, Dr. Wilks says, "Cancer cannot be inoculated; it does not run a definite course; and, indeed, has no qualities which deserve it to be considered as foreign to the organism of the body," and all attempts hitherto made to communicate Barsati to healthy animals have been attended by similar failures. Neither the human nor the equine form of the disease can be inoculated. I lay stress on this point, as I find observers on both sides pointing to exceptional cases of success attending their inoculation experiments. It is possible that there may exist an actual diseased state of system not yet developed, and where by local irritation in the attempt to inoculate with the discharge a nidus may be formed for the actual development of the disease, but then only as any other local irritant. Besides, as Dr. Tilbury Fox has observed, connective-tissue corpuscles, when altered by disease, have this power of communicating disease most markedly developed.

A large number of experiments have been made by me during the last two years on horses, ponies, and dogs, with fresh matter from Barsati growths, and no results were produced. Allowing, therefore, for the sake of argument, that Mr. Smith has found a new method of injection, which produces Barsati growths in healthy animals, does this prove that the parasites he has described are the cause of Barsati growths? Do we not know of connective tissue corpuscles, as altered in disease, producing degenerative changes in the tissues, and even leading in many cases to fatal results, as shown by Virchow, Dr. Tilbury Fox, and many other good authorities? Do we not know of similar corpuscles detected by Dr. Fleming in Delhi-Boil, and which were also inocuable, producing sores in many instances indistinguishable from Delhi-sores? The production of pus-cells from pressure, of granulation-cells due to altered nutrition, &c., show that cell changes are not always connected with parasitic action, but may be independent of it. We have detected several species of bacillus, vibria, and micrococcus in the scrapings from ordinary ulcers in India.

Given the extensive changes of the skin and subcutaneous connective tissue from long-standing ulceration in Barsati, and the surgeon has no difficulty in explaining a recrudescence of disease and its return in tissues originally attacked, by a process of cell-degeneracy, independently of the existence of fungi. The teachings of Huxley, Virchow, Uskoff, and others show that the theory of susceptibility and phlogogenous action of cell upon cell is not all nonsense; it is only out of fashion, because many, at the present time, know of no "infective" process other than that by germs and fungi. Dr. Wilks writes, "It is now known that a variety of morbid growths may be produced in the tissues, and that between the one which is styled cancer and that which is identical with healthy material, all grades may exist. They are but modifications of normal tissues, show only altered nutrition, and can by no means be regarded as foreign to the system." Health and disease are in the main easily distinguishable; but as we approach the frontier, so to speak, of either condition, the power of defining between what belongs to health and what to disease, becomes more and more difficult, the one apparently leading into the other through such an insensible series of gradations that it is impossible, as Professor Huxley holds, to say at any stage of the process—Here the line between health and disease must be drawn. On this view, false growths like Barsati and other tumors, are merely abnormal developments of normal growths.

Can we infer from the casual relationship of parasites with Barsati sores, that the former are necessarily the cause of the latter? With the fact recorded in the past regarding Barsati, it behoves both scientists and practitioners to look to their diagnosis, to improve if possible our knowledge of the clinical history of the disease so that its application to pathology should become both wider and more precise, for the precision of our results in practice must depend upon the perfection of our diagnosis. If the diagnosis of so serious a disease is to be arrived at by the arbitrary adoption of a single symptom, the presence of a vegetable fungus, the marvellous success of treatment in the hands of some practitioners, as compared with others, may be explained. While fully admitting the importance of a search after parasites as an aid to diagnosis, we fancy that on this, as on many other methods of research, too exclusive dependence should not be placed, as it is probable that fungi may develop under different disease-conditions, and under more circumstances than we at present suspect. That parasites should be found in many sores in India we readily grant, but we altogether dissent from the view of their being the cause of those sores.

In the case of certain cutaneous diseases in which organisms have been found, the latter appear to have the necessary connection with the diseased conditions, but such connection is wanting in the affection here referred to, viz., Barsati. We are not among those who reject all the vegetable theories of specific morbid processes. But the evidence in favour of the parasitic origin of Barsati, as well as of some other forms of sores met with in India, is not so strong as that concerning other diseases known to depend upon the growth and action of fungi. Drs. Smith and Fleming* previously maintained that fungi were seen in Delhi-boil; and Drs. Lewis and D. D. Cunningham,† have since shown that the development of these fungi in Delhi sores was due to the methods of preparing the specimen for microscopic examination, and may be seen in *healthy* tissues hardened in special media. And if the existence of the fungus is possible and frequent apart from a Barsati sore, why, it may be asked, is it likely that it is in any way a cause of it? It would be a begging of the whole question to infer that, because certain fungi grow and develop in Barsati sores, there is, therefore, evidence of cause and effect between the fungus and Barsati sore, and that the study of the disease has become superfluous.

It appears to me incorrect, therefore, to refer this disease to parasitic action, as has hitherto been traditionally done, at

* Army Medical Report, 1868-69.

† Report on 'Oriental-Sore,' or Lupus Endemicus, 1876.

least so long as we have not more definite clinical and pathological proofs than those we at present possess. Any change in the nomenclature of disease which is made without due consideration of facts of clinical medicine and pathology must surely hinder accuracy in diagnosis, and is therefore to be deprecated. Uskoff, Ponfick, Dr. Burdon Sanderson, Beale, Bennett, and others many years ago rejected an exclusively vegetable theory of morbid processes, which has not been controverted, and the idea of many normal cells capable of modification into the various forms of disease producing particles, commends itself at once as a view likely to reconcile many differences, and be acceptable to practical as well as inductive physiologists, pathologists, and clinicians.

Paget says: "From numerous observations made during the last few years it would appear that the characteristic cancer cells and nuclei may, like the corpuscles of pus, take their rise from pre-existing cells and nuclei of the texture or organ in which the new growth originates." This mode of origin of the structural elements of cancer was first pointed out by Virchow, and he has more particularly directed attention to the changes which take place in the connective tissues during the development and growth of cancer in it. Paget writes: "For the present I will only say that I think malignant tumors are local manifestations of some specific morbid states of the blood, and that in them are incorporated peculiar morbid materials which accumulate in the blood, and which their growth may tend to increase."

The anatomical elements of cancer are now known to have no special and peculiar characteristics, and they are believed to be as easily derivable from pre-existing tissues as are other non-specific morbid growths. Most physiologists, especially on the continent of Europe, now maintain that every structure, new or old, is formed by the proliferation of the pre-existing cells. Anatomists have also done much to shake the fixedness of our methods of healing by finding evidence to show that what appear superficially different forms of morbid products are, in reality, different grades.

(To be Continued).

